# *Ecklonia cava* Extract and Its Derivative Dieckol Promote Vasodilation by Modulating Calcium Signaling and PI3K/AKT/eNOS Pathway in In Vitro and In Vivo Models

**DOI:** 10.3390/biomedicines9040438

**Published:** 2021-04-19

**Authors:** Yu-An Lu, Jun-Geon Je, Jin Hwang, You-Jin Jeon, BoMi Ryu

**Affiliations:** 1 Department of Marine Life Science, Jeju National University, Jeju 63243, Korea; annie.lu1213@gmail.com (Y.-A.L.); wpwnsrjs@naver.com (J.-G.J.); ghkdwls9280@naver.com (J.H.); 2 Marine Science Institute, Jeju National University, Jeju 63333, Korea

**Keywords:** *Ecklonia cava*, dieckol, NO production, calcium transit, M3 muscarinic acetylcholine receptor, endothelial cell, vasodilation, zebrafish

## Abstract

Nitric oxide (NO), an endothelial-derived relaxing factor synthesized by endothelial nitric oxide synthase (eNOS) in endothelial cells, enhances vasodilation by modulating vascular tone. The calcium concentration critically influences eNOS activation in endothelial cells. Thus, modulation of calcium-dependent signaling pathways may be a potential therapeutic strategy to enhance vasodilation. Marine algae reportedly possess protective effects against cardiovascular disorders, including hypertension and vascular dysfunction; however, the underlying molecular signaling pathways remain elusive. In the present study, we extracted and isolated dieckol from *Ecklonia cava* and investigated calcium transit-enhanced vasodilation. Calcium modulation via the well-known M3 muscarinic acetylcholine receptor (AchM3R), which is linked to NO formation, was investigated and the vasodilatory effect of dieckol was verified. Our results indicated that dieckol effectively promoted NO generation via the PI3K/Akt/eNOS axis and calcium transients influenced by AchM3R. We also treated *Tg(flk: EGFP)* transgenic zebrafish with dieckol to assess its vasodilatory effect. Dieckol promoted vasodilation by enlarging the dorsal aorta diameter, thus regulating blood flow velocity. In conclusion, our findings suggest that dieckol modulates calcium transit through AchM3R, increases endothelial-dependent NO production, and efficiently enhances vasodilation. Thus, *E. cava* and its derivative, dieckol, can be considered as potential natural vasodilators.

## 1. Introduction

Under physiological conditions, the vascular endothelium plays a critical role in regulating vascular tone and blood pressure (BP) by generating vasodilating and vasoconstricting factors to suppress cardiovascular disease (CVD) (e.g., hypertension and acute coronary syndromes) [1,2,3]. Nitric oxide (NO) is a well-known vessel-relaxing factor produced from L-arginine by endothelial nitric oxide synthase (eNOS) in the presence of oxygen and the cofactors Ca^2+^ and calmodulin [4]. A previous study indicated that genetically deficient eNOS mice are hypertensive, with lower circulating NO levels, thus indicating the critical role of eNOS and NO in CVD [5,6]. Cytosolic [Ca^2+^] ([Ca^2+^]_cytol_) can either increase because of an cytosol influx through specific calcium channels expressed on cellular surfaces or by a release from intracellular stores such as the endoplasmic reticulum (ER) [7]. Furthermore, ER [Ca^2+^] has long been proposed as a critical factor for regulating eNOS activity, resulting in vasodilation [6]. Calcium homeostasis is affected by acetylcholine (Ach) or vascular endothelial growth factor (VEGF), which plays a major role in regulating vasodilation by increasing [Ca^2+^]_cytol_ levels via calcium influx, which further promotes the expression of downstream proteins such as eNOS [8,9]. Furthermore, the increased expression of Akt directly phosphorylates eNOS, thereby increasing its binding to intracellular calmodulin, activating eNOS, and promoting NO release [8,10]. Thus, the increase in NO production effectively modulates vascular tone to prevent cardiovascular diseases.

*Ecklonia cava* (E. cava, EC) has revealed different biological activities, including antioxidant, anti-inflammatory, attenuation of endothelial cell dysfunction, and antihypertension, in numerous studies [11,12,13,14]. Son et al. indicated that EC ethanol extract (ECE) significantly alleviates blood pressure (BP) in a mouse model of hypertension. Notably, a previous study revealed that ECE regulates BP by inhibiting angiotensin-converting enzyme (ACE) in a rat model of hypertension [15,16]. ACE elevates BP by converting the hormone angiotensin I to the progressive vasoconstrictor angiotensin II [17]. Furthermore, based on the superior antihypertensive effects of ECE, dieckol (DK), a polyphenolic compound present in ECE, has been suggested as one of the bioactive components responsible for the potential ACE inhibitory activity [18,19]. Moreover, DK reportedly improves BP control in hypertensive in vivo models via the ACE inhibitory activity for managing hypertension [19]. However, investigations on the antihypertensive effects of ECE and DK have primarily focused on ACE inhibition; [Ca^2+^] homeostasis in vascular endothelial cells, a crucial feature of vasodilation that could improve vascular health and function, needs to be evaluated. Therefore, in the present study, we investigated the vasodilatory properties of ECE and DK associated with [Ca^2+^] modulation.

## 2. Materials and Methods

### 2.1. Reagents

Dulbecco’s modified Eagle’s medium (DMEM) and penicillin/streptomycin (P/S) were purchased from GIBCO (Grand Island, NY, USA); fetal bovine serum (FBS) was obtained from Merck (Sacramento, CA, USA); dimethyl sulfoxide (DMSO) and 3-(4-5-dimethyl-2yl)-2-5-diphynyltetrazolium bromide (MTT) were purchased from Sigma Co. (St. Louis, MO, USA); NO production was measured by DAF-FM-DA (4 amino-5-methylamino-2′, 7′-difluorescein diacetate; (Sigma-Aldrich, St. Louis, MO, USA). Calcium levels were detected using fluo-4-AM dye (1-[2-amino-5-(2,7-difluoro-6-hydroxy-3-oxo-9-xanthenyl)phenoxyl]-2-(2-amino-5-methylphenoxy) ethane-N, N, N, N′-tetraacetic acid, pentaacetoxymethyl ester) (Thermo Fisher Scientific, Waltham, MA, USA). Atropine, a specific acetylcholine receptor antagonist, was purchased from Sigma-Aldrich (St. Louis, MO, USA).

### 2.2. ECE Preparation and DK Isolation

In brief, the method for preparing ECE and DK was as follows: EC was collected in April on Jeju Island, South Korea. First, EC was washed with running water to remove salt, sand, and epiphytes attached to the surface. Then, it was lyophilized and ground into a dry powder, which was extracted with 80% ethanol at room temperature for 24 h. The isolation of DK was performed according to a previously published method [20]. The BUCHI pure chromatography system (BUCHI, Pure C-850 FlashPrep, Flawil, Switzerland) was used for DK separation. Chromatography was performed on Agilent Technologies 1220 Infinity II LC with a column (poroshell 120 C18, 4.6*100 mm, 4µm). The mobile phase consisted of A; DW (+0.1% Formic acid), B; MeOH (+0.1% Formic acid) as followed: (0 min A; 63% B; 37%, 0–10 min A; 45% B; 55%, 10–12 min A; 63% B; 37%, 12–20 min A; 63% B; 37%). The gradient elution was performed as follows: the flow rate was 0.4 mL/min, and the injection volume was 1 mL. Detection was performed at UV length 230 nm. (Supplementary Materials, Figure S1 illustrates the HPLC chromatography analysis data for the isolated DK). 

### 2.3. Measurement of Cell Viability and NO Production

Human cardiovascular endothelial cells (EA.hy926 cell line, ATCC, Manassas, VA, USA) were grown in DMEM with 10% FBS and 1% P/S mixture. The cells were grown at 37 °C in a humidified incubator with 5% (*v*/*v*) CO_2_. The cell passages from 2–5 were used for experimentation.

To evaluate the impact of ECE and DK on cell viability, EA.hy926 cells were employed using the colorimetric MTT assay method as described by Arutyunyan et al. [21]. These EA.hy926 cells, at a density of 1 × 10^5^ cells/mL, were seeded in 96-well plates. The cells were treated with ECE (3, 10, 30, and 100 µg/mL) and DK (4, 13, 40, and 134 µM), with three replicates performed for each concentration. After 24 h, the medium was replaced with 50 µL of MTT solution and incubated for 2 h at 37 °C. The insoluble formazan was dissolved in 100 μL of DMSO at room temperature and shaken overnight. The absorbance was measured at 540 nm using a microplate reader (Synergy HT, BioTek Instruments, Winooski, VT, USA). Cell viability was normalized as a percentage of the control (without treatment with ECE and DK treatments). The supernatants (100 µL) in EA.hy926 cells were transferred to a reaction volume of 100 μL. Intracellular NO production was detected using DAF-FM-DA (Sigma-Aldrich, St. Louis, MO, USA). Following treatment, the cells were washed three times with phosphate-buffered saline (PBS) and incubated with a final concentration of 5 µM for 30 min at room temperature in the dark. Fluorescence intensity was measured at an excitation wavelength of 495 nm and an emission wavelength of 515 nm using a microplate reader.

### 2.4. Measurement of Cytosolic [Ca^2+^] Levels

Extracellular Ca^2+^ levels refer, by definition, to the calcium ion concentration in the cytosol ([Ca^2+^]_cytol_). To detect the [Ca^2+^]_cytol_ levels, the sensitive probe Fluo-4 was dissolved in the physiological salt solution (PSS), consisting of 140 mM NaCl, 5.9 mM KCl, 1.4 mM MgCl_2_·6H_2_O_2_, 10 mM HEPES, 11.5 mM glucose, 1.2 mM NaH_2_PO_4,_ 5mM NaHCO_3_, and 1.8 mM CaCl_2_, at pH 7.4 with NaOH. Then, EA.hy926 cells were seeded in 96-well plates overnight until they reached 80% confluency. Next, 1× Fluo-4 was added and incubated for 30 min at 37 °C in the dark. Next, the cells were rinsed twice with 1 × PBS, with 50 µL PBS added to each well. After measuring the intensity of fluorescence for 10 s at a time interval of 1 s, cells were directly treated with 1×PSS and different sample concentrations dissolved in 0.1% bovine serum albumin (BSA). Fluorescence detection was continuously performed for another 50 s at a time interval of 1 s, and changes in calcium levels were detected using the Gen5 3.04 software (Synergy HT, BioTek Instruments, Winooski, VT, USA). Moreover, to investigate whether DK activated the downstream pathways by connecting with AchM3R, cells were pretreated with the specific antagonist atropine (AT) at 100 µΜ and incubated for 1 h. Next, 1 × Fluo-4 fluorescent dye was added to the cells, followed by incubation for another 30 min at 37 °C in the dark. Further steps were identical to those performed for measuring [Ca^2+^]_cytol_ levels.

### 2.5. Western Blot Analysis

The EA.hy926 cells were harvested and lysed for Western blot analysis. After centrifugation of cells at 12,000 rpm for 20 min, the protein content of supernatants was evaluated using a BSA protein assay kit (Bio-Red, Hercules, CA, USA). Sodium dodecyl sulfate-polyacrylamide gel electrophoresis (SDS-PAGE) (10%) and proteins were transferred onto a nitrocellulose membrane. The membranes were incubated overnight at 4 °C with the following primary antibodies: anti-β-actin (sc-47778, Santa Cruz Biotechnology, CA, USA; 1:1000), anti-*p*-Akt (sc-377556, Santa Cruz Biotechnology, CA, USA; 1:1000), anti-*p*-eNOS (#9571S, Cell Signaling Technology, Danvers, Massachusetts; 1:1000), anti-*p*-PI3K (#17366S, Cell Signaling Technology, Danvers, Massachusetts; 1:1000), and dissolved in 5% skim milk. The immunoblots were incubated for another 2 h at room temperature with specific secondary antibodies. The bands were detected using a chemiluminescent substrate (Cyanagen Sri, Bologna, Italy) and visualized on a film using a FUSION SOLO Vilber Lourmat system (FUSION, Paris, France). The band intensity was calculated using ImageJ software 1.50i software (National Institutes of Health, Bethesda, MD, USA).

### 2.6. Molecular Docking of DK with AchM3R

The 3D structure of AchM3R (PDB: ID 5ZHP) was obtained from the Protein Data Bank in pdb format. All heteroatomic molecules were excluded from the file using Accelrys Discovery Studio (DS) 3.0 (Inc., San Diego, CA, USA). To prepare the docking procedure, we performed the following steps: (1) conversion of the 2D structure into a 3D structure, (2) calculation of charges, and (3) addition of hydrogen atoms using the CDOCKER docking program. ChemDraw software was used to draw the 2D structures of these compounds and convert them into 3D structures, optimized, and saved in the pdb file format by Spartan’14 v.1.1.2 Irvine, California, USA [16]. The compounds were converted to PDBQT format using AutoDock 4.2 software (Scripps Research, San Diego, CA, USA).

### 2.7. Vascular Response of the Transgenic Zebrafish Tg(flk:EGFP) Model

The transgenic zebrafish Tg(flk:EGFP) was used to perform an in vivo investigation [22]. Fish were housed in 3 L tanks (aquatic habitats). The zebrafish facility contained buffered water (pH 7.5) at 28.5 °C. Fertilized eggs were collected from the bottom of the tank in an automatic circulation culture system (ESEN, Beijing, China), maintained at a temperature of 28.5 °C, pH 7.5, dissolved oxygen 7.0, conductivity 800 µS, containing methylene blue. The eggs were placed in Petri dishes after thorough washing in the system water and then transferred to the incubator. For experiments, the larvae were first maintained in 12-well plates containing egg water (reverse osmosis water containing 60 mg sea salt per liter of water (pH 7.5)). After sample treatment, the number of larvae was checked daily. For DK, an in vivo toxicity test was performed using the zebrafish model as follows. The test was based on the exposure of newly fertilized zebrafish eggs to the test sample for up to 120 h; 15 eggs per treatment (three replicates) were selected and distributed in 12-well microplates. The test was initiated with newly fertilized eggs exposed to 4, 13, 40, and 134 µM of DK and run for 144 h. Embryos were observed for up to 144 h under a stereomicroscope (magnification used in the stereomicroscope for observations was 4×). The vasodilatory effect of DK in larvae was demonstrated as follows: Larvae were treated with 4, 13, 40, and 134 µM of DK at three days post-fertilization (3 dpf). After six days of treatment, the larvae were photographed using a fluorescence microscope; 4× magnification was used to capture vessels in the whole body. The fluorescence intensities of whole-body vessels were determined using Gen5 3.04 (Synergy HT, BioTek Instruments, Winooski, VT, USA) and then averaged. The fluorescence intensities of the images (4× magnification) were analyzed using ImageJ software.

CTOF (corrected total object fluorescence) = Integrated Density − (Area of selected object × mean fluorescence of background readings)

The dorsal aorta (DA) was used to measure cardiac parameters, including heartbeat, mean linear flow, arterial pulse, and diameter. Images of vessel diameter were captured using a fluorescence microscope (Gen5 v.3.04 software, Synergy HT, BioTek Instruments, Winooski, VT, USA), and the mean value was analyzed and calculated using ImageJ software. Moreover, the arterial pulse (beats per minute), mean blood flow velocity (μm/s), and blood flow (nL/s) were determined using a pre-recorded video at 120 frames per second (fps) for 1 min using the Gen5 v.3.04 software (Synergy HT, BioTek Instruments, Winooski, VT, USA). Then, the MicoZebraLab application from ViewPoint (v.3.4.4, Lyon, France) was used to evaluate the cardiovascular parameters mentioned above.

### 2.8. Statistical Analysis

All data were analyzed in triplicate, and the results are expressed as the mean ± standard deviation. Statistical analysis was performed using one-way ANOVA with Dunnett’s post hoc test, using GraphPad Prism 5.0 (GraphPad Software, La Jolla, CA, USA) to determine significant differences from the blank, with * *p* < 0.05, ** *p* < 0.01, and *** *p* < 0.001 considered significant.

## 3. Results

### 3.1. Effect of ECE and DK on Intracellular NO Production in EA.hy926 Cells

**Figure 1:**
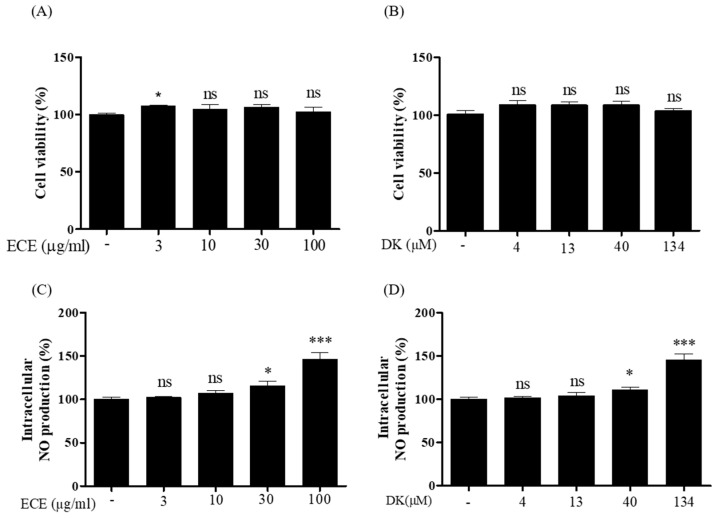
Cell viability analysis using EA.hy926 cells treated with (**A**) ECE (0, 3, 10, 30, and 100 µg/mL) and (**B**) DK (0, 4, 13, 40, and 134 µM). Intracellular NO production by (**C**) ECE and (**D**) DK. Results are expressed as the mean ± standard deviation (S.D.) of three independent experiments. * *p* < 0.05, *** *p* < 0.001 significantly different compared with the control group. EC, *E. cava* extract; DK, Dieckol; NO, nitric oxide; ns, not significant; -: no sample treatment (control)

For ECE and DK, the viability of EA.hy926 cells was investigated using different concentrations of ECE (3, 10, 30, and 100 µg/mL) and DK (4, 13, 40, and 134 µM). As shown in Figure 1A,B, investigated ECE and DK concentrations showed no significant toxic effects when compared with the control. Nontoxic dosages were used in subsequent experiments. Intracellular NO production by ECE and DK in EA.hy926 cells is illustrated in Figure 1C,D. A significant increase in NO production was observed at 30 and 100 µg/mL of ECE and 40 and 134 µM of DK. As NO levels can be induced by specific ECE and DK concentrations, the possible vasodilative mechanisms in endothelial cells were investigated.

### 3.2. Effect of ECE and DK on the Phosphorylation of the PI3K/Akt/eNOS Pathway in EA.hy926 Cells

**Figure 2:**
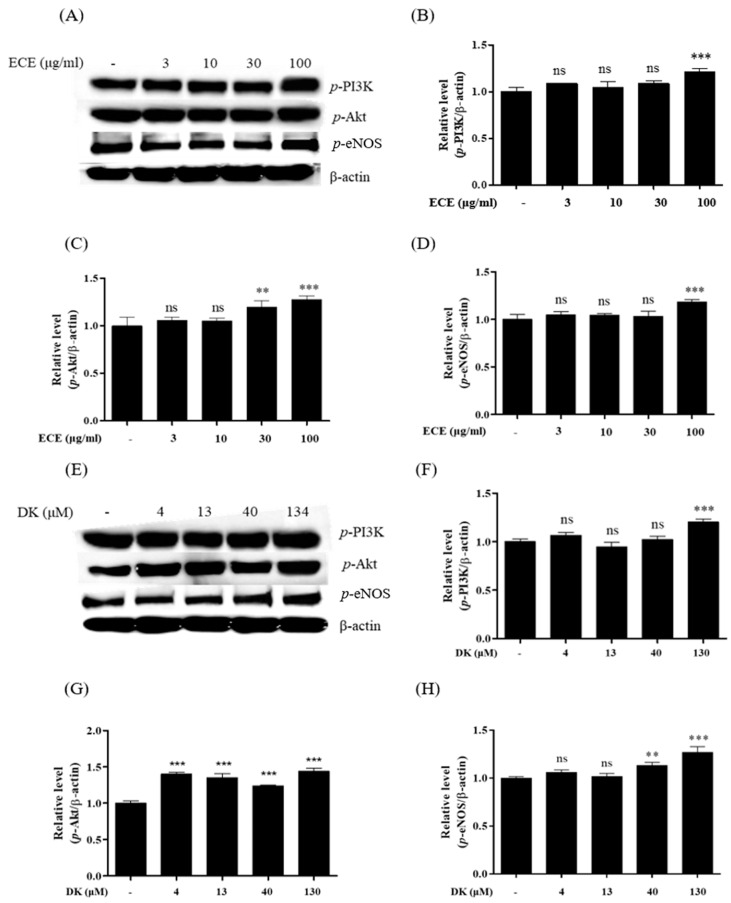
Evaluation of vasodilation-related protein expression. Examples of Western blot analysis, (**A**,**E**). The protein expressions of ECE treatment on (**B**) phosphorylated-PI3K *(p*-PI3K), (**C**) *p*-Akt, and (**D**) *p*-eNOS; the protein expressions of (**F**) *p*-PI3K, (**G**) *p*-Akt, and (**H**) *p*-eNOS following DK treatment. The protein bands were ultimately developed and photographed with the FUSION Solo Vilber Lourmat system. Quantitative data were analyzed using ImageJ software. Results are expressed as the mean ± standard deviation (S.D.) of three independent experiments. * *p* < 0.05, ** *p* < 0.01, *** *p* < 0.001 significantly different compared with the control group. EC, *E.Cava* extract; DK, Dieckol; PI3K, phosphoinositide 3-kinases; eNOS, endothelial nitric oxide synthase; -: no sample treatment (control)

In the endothelium, phosphorylation of PI3K and Akt promotes eNOS activity, further enhancing NO production [23]. Thus, to verify the NO production pathway induced by ECE and DK in endothelial cells, the protein levels of p-PI3K, p-Akt, and p-eNOS were investigated. Western blot plots following ECE and DK treatments are shown in Figure 2A,E. Compared with the control (Figure 2B), *p*-PI3K was significantly increased at 100 µg/mL ECE. Additionally, an effective increase was observed at 134 µM DK (Figure 2F). Activated *p*-PI3K can further stimulate downstream *p*-Akt expression [24]. As shown in Figure 2C, *p*-Akt expression was significantly increased in 30 and 100 µg/mL by a concentration-dependent manner. Besides, we observed that the phosphorylation of Akt could be effectively enhanced by DK concentrations employed (Figure 2G). Moreover, the increasing trend of *p*-eNOS was only observed at ECE 100 µg/mL compared to control in Figure 2D. And also, DK treatments (40 and 130 μM of DK) remarkably enhanced the *p*-eNOS expression (Figure 2H). These findings revealed the potential of ECE and DK to promote key proteins of the PI3K/Akt/eNOS pathway, which can lead to NO production in EA.hy926 cells.

### 3.3. Effect of ECE and DK on [Ca^2+^]_cytol_ Levels in EA.hy926 Cells

**Figure 3:**
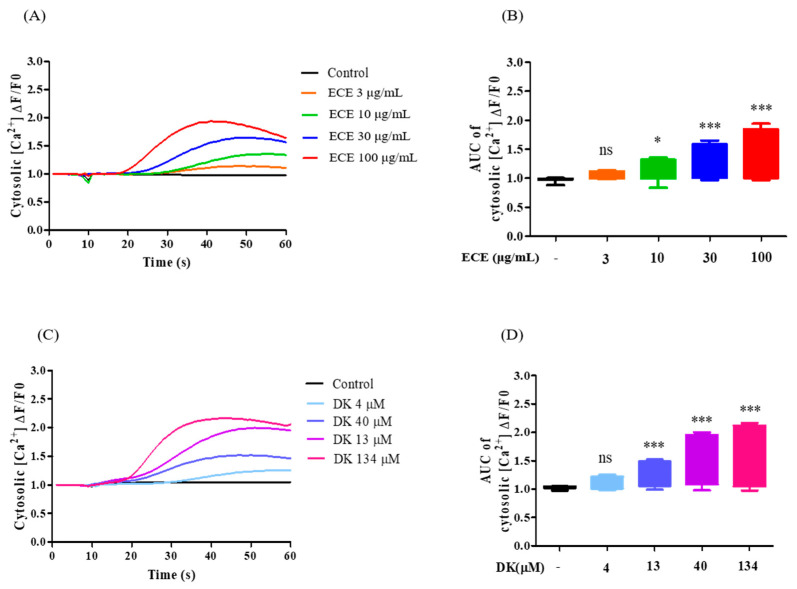
Quantification of [Ca^2+^]_cytol_ levels following treatment with different ECE and DK concentable 926. cells. In the absence of [Ca^2+^]_cytol_ using the Fluo-4 calcium indicator, (**A**) traces and (**B**) box plot representation of [Ca^2+^]_cytol_ levels under ECE treatments (3, 10, 30, and 100 µg/mL); The absence of [Ca^2+^]_cytol_ levels in cell (**C**) traces and (**D**) box plot representation of [Ca^2+^]_cytol_ levels in response to the addition of DK (0, 4, 13, 40, and 134 µM). Results are expressed as the mean ± standard deviation (S.D.) of three independent experiments. * *p* < 0.05, ** *p* < 0.01, *** *p* < 0.001 significantly different compared with the control group. EC, *E.Cava* extract; DK, Dieckol; ns, not significant; AUC, area under the curve; [Ca^2+^]_cytol_, calcium level in the cytosol.

In the present study, the [Ca^2+^]_cytol_ level represents calcium levels in the cytosol, which can be influenced by calcium influx (influx of extracellular calcium into the cytosol via voltage-dependent calcium channel (VDCC)) and the activation of specific receptors such as AchM3R. To investigate the regulatory effects of ECE and DK, the concentrations of [Ca^2+^]_cytol_ were measured. All samples were treated for 10 s and continuously traced for 60 s. As shown in Figure 3A, the time curve revealed that the different ECE concentrations induced a dose-dependent increase in [Ca^2+^]_cytol_ levels. The maximum [Ca^2+^]_cytol_ level was observed in the 100 µg/mL ECE group. Additionally, the area under the curve (AUC) of [Ca^2+^]_cytol_ at each concentration is compared in Figure 3B. The results indicated that the effective dosage of ECE was 10 µg/mL, and the optimal concentration was 100 µg/mL. Furthermore, we evaluated the ability of DK to detect [Ca^2+^]_cytol_ levels. As shown in Figure 3C, DK treatment resulted in a significant increase in [Ca^2+^]_cytol_, with an AUC value approximately two times higher in [Ca^2+^]_cytol_ at 134 µM DK. Thus, 134 µM DK (DK134) was selected for further investigations to assess the role of each receptor in elevated vasodilatory [Ca^2+^]_cytol_ levels induced by DK in EA.hy926 cells. Collectively, ECE and DK effectively increased [Ca^2+^]_cytol_ levels; in particular, the levels of [Ca^2+^]_cytol_ were higher following DK treatment than those induced by ECE at equivalent concentrations.

### 3.4. Molecular Docking Study of DK and AchM3R

**Figure 4:**
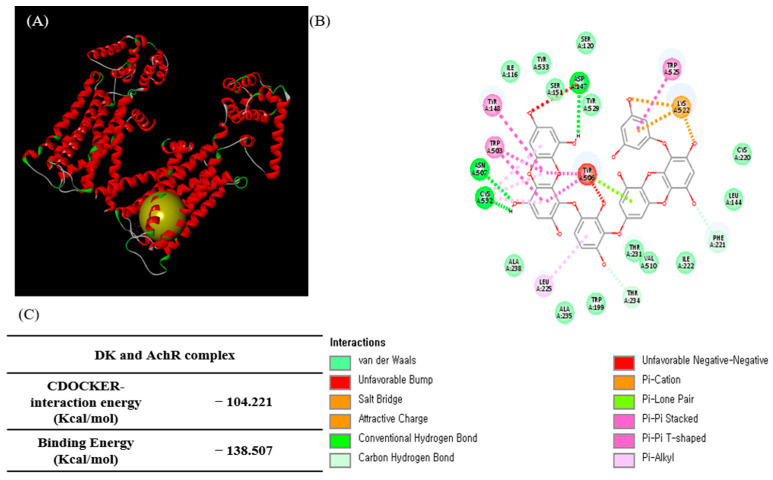
Computational prediction of the AchM3R and docking stimulation with DK. (**A**) 3D diagram and (**B**) 2D diagram of AchM3R and DK complex. (**C**) Results of the docking experiments with DK with AchM3R. DK, Dieckol; AchM3R, muscarinic M3-receptor.

After demonstrating that ECE and DK markedly increased [Ca^2+^]_cytol_ levels, we further investigated the DK structure based on a computational analysis of the role of DK in the transit of vasodilatory [Ca^2+^]_cytol_ in EA.hy926 cells. To confirm the interaction of DK and AchM3R, the binding ability was determined via molecular docking studies. Computational prediction of AchM3R residues interacting with DK and the 3D structure of the complex are shown in Figure 4A. The 2D diagram of the interaction between DK and the amino acid residues of nearby active sites is shown in Figure 4B. The docking model revealed electrostatic interactions and a network of hydrogen bonds in the complex [25]. DK docking results with AchM3R generated hydrogen bond interactions with Asp347, Asn A507, and Cys A532. Moreover, van der Waals contacts were observed with Try529, Ser120, ser151, Try533, Ile116, Ala238, Ala235, Trp199, Thr231, Val510, Ile222, Leu144, and Cys220, resulting in unfavorable bump interactions with Tyr506.

Additionally, the lower the energy value, the higher the docking scores. The CDOCK interaction energy was −104.221 kcal/mol, and the binding energy was −138.507 kcal/mol for DK and AchM3R (Figure 4C). Overall, the computational analysis revealed that DK affected AchM3R and might stimulate the downstream pathway (e.g., calcium transit) by binding with this receptor. Therefore, specific antagonists of AchM3R were used to investigate related mechanisms.

### 3.5. DK Induced [Ca^2+^]_cytol_ Transient via AchM3R

**Figure 5:**
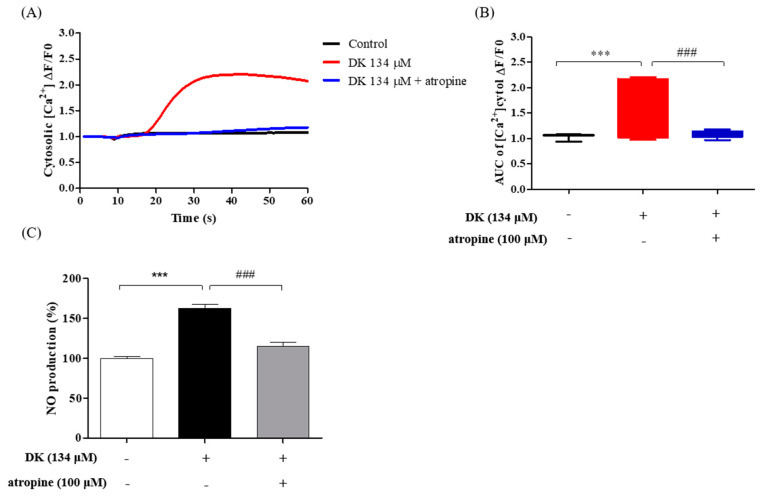
Influence of a specific antagonist on [Ca^2+^]_cytol_ levels in EA.hy926 cells treated with DK. (**A**) Traces and (**B**) box plots indicating [Ca^2+^]_cytol_ levels in response to treatment with DK (134 μM) and antagonist. (**C**) Effect of DK on NO production in EA.hy926 cells pretreated with atropine. The NO levels were detected by adding 10 μM of 4 amino-5-methylamino-2′, 7′-difluorescein diacetate (DAF-FM-DA). Results are expressed as the mean ± standard deviation (SD) of three independent experiments, *** *p* < 0.001 significantly different compared with the control group; ### *p* < 0.01 significantly different compared with DK only group. DK, Dieckol; ns, not significant; AUC, area under the curve; [Ca^2+^]_cytol_, calcium level in the cytosol; NO, nitric oxide; -: no sample treatment (control)

Furthermore, we assumed that the calcium level induced by DK was affected by AchM3R. To verify our hypothesis, the specific antagonist, atropine, was employed. A rapid increase was observed only in the DK134 treatment group when compared with the control group (Figure 5A). Additionally, the [Ca^2+^]_cytol_ levels of the DK134+AT group showed a significant decrease when compared with the DK134 only group (Figure 5B). Next, we investigated whether the vessel-relaxing factor, NO, was influenced by the receptor blocker and DK. The results indicated that the atropine significantly inhibited intracellular NO production and that DK could induce intracellular NO production through AchM3R (Figure 5C). Overall, the results demonstrated that a DK treatment induced vasodilation in vascular endothelial cells by elevating vasodilators, i.e., [Ca^2+^]_cytol_ levels and intracellular NO in endothelial cells by activating AchM3R.

### 3.6. DK-Promoted Vasodilation in Zebrafish Transgenic (flk: EGFP) Model

**Figure 6:**
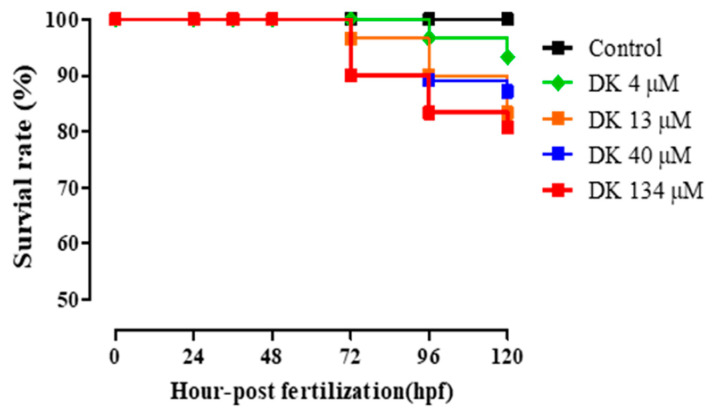
The survival rate of the zebrafish embryos under DK treatment. The test was based on the exposure of newly fertilized zebrafish eggs to 4, 13, 40, and 134 µM of DK for up to 120 h (*n* = 15 per treatment, three replicates). Embryos were observed at each time point under the stereomicroscope (magnification used in the stereomicroscope for observations was 4×). DK, Dieckol.

Zebrafish (*Danio rerio*) is an important vertebrate model for analyzing blood and vascular development [26]. Transgenic zebrafish (flk: EGFP) express the fluorescent protein EGFP in vascular endothelial cells (green fluorescence) under the control of the flk promoter. Furthermore, fluorescently labeled transgenic strains of the endothelium are available for investigating vessel behaviors such as dilation or contraction under test sample treatment [27]. Therefore, Tg (flk: EGFP) is considered a feasible model for assessing cardiovascular diseases [28]. To investigate whether DK could promote vasodilation in the zebrafish model, Tg (flk: EGFP) zebrafish was employed. The toxicity of different DK concentrations was examined in zebrafish embryos. A survival rate exceeding 80% in the zebrafish experiment was considered nontoxic, which can be used for further investigations [29]. As shown in Figure 6, the various DK concentrations showed no toxicity in zebrafish embryos at 120 hpf (hours post-fertilization).

**Figure 7:**
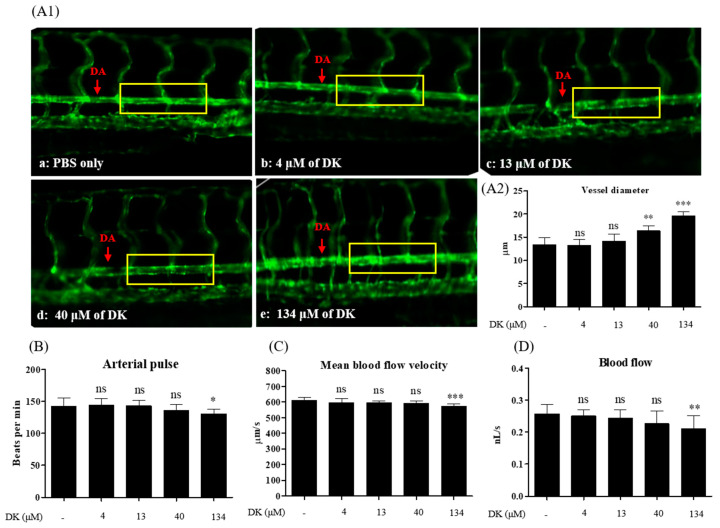
Cardiovascular parameters measured at the dorsal aorta (DA) of 3 dpf Tg(flk:EGFP) transgenic zebrafish. (**A1**) Images of the DA captured by fluorescence microscope (20×) (**a**–**e**) Vasodilation observed by treatment with DK. (**a**) PBS (Control); (**b**) 4 μM of DK; (**c**) 13 μM of DK; (**d**) 40 μM of DK; (**e**) 134 μM of DK. (**A2**) Evaluation of DA vessel diameter. (**B**) Arterial pulse (beats per minute); (**C**) Mean blood flow velocity (μM/s); (**D**) Blood flow (nL/s) were measured from *n* = 6 larvae per group. The yellow box represented the area used for evaluation. Results are expressed as the mean ± standard deviation (S.D.). * *p* < 0.05, ** *p* < 0.01. ^##^ *p* < 0.01. *** *p* < 0.001 significantly different compared with the control group. dpf, days post-fertilization; ns, not significant; -: no sample treatment (control)

Further, we provided direct evidence by measuring the DA diameter to confirm the vasodilatory effects induced by DK treatment in vivo. As shown in Figure 7(A2), except for 4 and 13 μM DK, the diameter of DA in DK treatment groups (40 and 134 μM) was significantly increased when compared with the control (PBS only). Other blood flow parameters, such as arterial pulse (Figure 7B), mean linear flow (Figure 7C), and blood flow (Figure 7D), gradually decreased in a dose-dependent manner when compared with the control. Consistent with the in vitro data, it can be suggested that DK enhances vasodilation and promotes blood flow in the zebrafish model.

## 4. Discussion

In recent years, endothelial dysfunction has been indicated by the low bioavailability of NO with a relevant negative impact on the NO/cGMP pathway [30]. The loss of NO/cGMP signaling may further cause smooth muscle cell contraction resulting in hypertension and other vessel-related disorders [31]. Also, the previous study demonstrated that impaired endothelial-dependent vasodilation and excessive oxidative stress are closely related [32]. Taddei, et al., indicated that impaired NO production can precede endothelial dysfunction in human hypertension [33]. Accumulated evidence has demonstrated that NO is a potent vasodilator for maintaining vascular homeostasis; when NO is released from endothelial cells and diffuses into the smooth muscle cells, a series of biochemical reactions occurs in vessel relaxation [34]. Recently, natural resources such as plant and marine algae extracts and their bioactive metabolites have emerged as potential alternatives for therapeutic application in cardiovascular diseases that affect humans via regulating endothelial-dependent NO relaxing [35]. Previously, EC was reported to have alleviated BP significantly in the renal arteries of hypertensive in vivo models, implicating ACE inhibitory activity [14,16,17]. *E. cava* and its constituent, DK, are postulated to block the production of vasoconstrictor angiotensin II by inhibiting ACE. This enzyme converts angiotensin I to angiotensin II to exert beneficial effects on the homeostatic regulation of BP. These findings are consistent with our results. In the present study, we demonstrated the mechanism underlying the effects of EC and DK on endothelium-derived relaxing factors (NO and [Ca^2+^]) to enable blood vessel changes and adequate blood flow. There are two main mechanisms of vasodilation that have been reported: (i) the calcium-independent (PI3K/Akt/eNOS) pathway and (ii) the calcium-dependent (Ca^2+^/calmodulin) pathway [36,37]. The PI3K/Akt/eNOS pathway demonstrated a slow but sustained increase. In contrast, Ca^2+^/calmodulin-dependent activation is a rapid and a relatively short-lasting mechanism that activates eNOS expression [38]. eNOS activation can be modulated in a more prolonged pattern by phosphorylation at Ser1177 [39].

Additionally, PI3K and protein kinase B (Akt) are closely associated [40]. We observed that ECE (30 and 100 μg/mL) and DK (40 and 134 μM) significantly increased NO production. Thus, we speculated that ECE and DK enhanced NO production via the PI3K/Akt/eNOS axis or [Ca^2+^]/calmodulin pathways. Indeed, the main proteins (PI3K, Akt, and eNOS) showed a concentration-dependent effect during ECE and DK treatments. Although similar PI3K and Akt levels were observed following ECE and DK treatments, the expression of eNOS under ECE treatment was lower than that observed following the DK treatment. Additionally, we hypothesized that DK could enhance [Ca^2+^]_cytol_ transit and facilitate [Ca^2+^]-calmodulin complex formation by increasing eNOS. As expected, we observed that both ECE and DK significantly increased [Ca^2+^]_cytol_ levels. Moreover, compared with ECE, DK treatment revealed higher [Ca^2+^]_cytol_ levels. In theory, a decreasing stabilization stage, usually characterized by the presence of less intense and less frequent oscillations, follows the peak phase [41]. However, within 60 s, the aforementioned trend was not observed. Thus, a longer detection time is required for future experiments. After revealing that DK can effectively increase [Ca^2+^]_cytol_ levels, we further focused on how DK regulates calcium transit via the transmembrane receptor (AchM3R).

**Figure 8:**
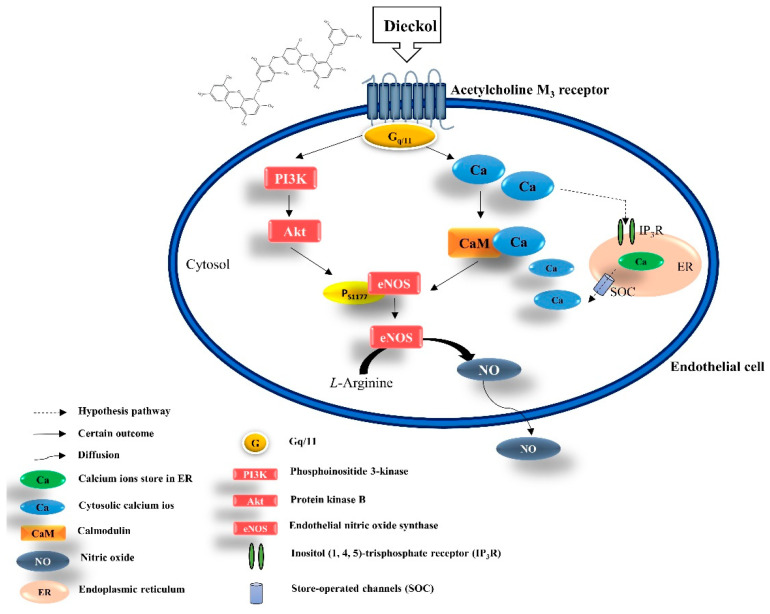
A schematic represented the potential endothelial-dependent vasodilation mechanisms of DK. DK possess potential vasodilatory effects by upregulating the expression of PI3K/Akt/eNOS and enhancing the [Ca^2+^]_cytol_ transit via AchM3R, resulting in NO formation in endothelial cells.

Muscarinic acetylcholine receptors belong to the G-protein-coupled receptor (GPCR) superfamily, a critical biological signaling protein [42]. There are five muscarinic receptor subtypes (M1–M5), consisting of seven transmembrane helices connected by six alternating extracellular and intracellular loops [43]. The AchM3 receptor selectively interacts with the Gq/11 proteins and mediates several physiological functions, including stimulation of phospholipid metabolism, calcium release, and smooth muscle contraction [43]. Due to its unique characteristics, the AchM3 receptor has been targeted for treating different types of diseases, including obesity [44], heart failure [45], and pulmonary hypertension [46]. Furthermore, once the AchM3 receptor is activated, downstream mechanisms are initiated with the conversion of phosphatidylinositol (PIP2) to inositol triphosphate (IP3) and diacylglycerol (DAG). IP3 interacts with the IP3 receptor on the ER to liberate calcium ions. The increased cytosolic calcium concentration, due to the emptying of the ER calcium stores, causes calcium ion influx by store-operated calcium entry (SOC) [6]. Free calcium ions bind with calmodulin and further stimulate the phosphorylation of eNOS, resulting in NO formation. As expected, the cells preincubated with atropine completely eliminated the calcium oscillations induced by DK treatment. Moreover, the concentration of intracellular NO was significantly suppressed by atropine treatment. Therefore, we suggest that ECE and DK possess potential vasodilatory effects by upregulating the expression of PI3K/Akt/eNOS and enhancing the [Ca^2+^]_cytol_ transit via AchM3R, resulting in NO formation in endothelial cells (Figure 8).

Based on the in vitro findings, we further confirmed the vasodilation property in the zebrafish model. Zebrafish are considered to be a superior investigational model owing to their outstanding advantages, such as the optical clarity of embryos, rapid development, and organ systems and gene functions being similar to those of humans [27]. The zebrafish model previously revealed several essential insights into vascular structure development and helped verify underlying molecular mechanisms [47]. The DA, a critical trunk artery, has been used to evaluate different cardiovascular parameters, such as mean linear flow, arterial pulse, and vessel diameter [48]. The diameter of DA in transgenic zebrafish was considered to be direct evidence when assessing sample treatment. As expected, compared with the control, treatment with 40 and 134 μM DK significantly increased the DA diameter. Reportedly, the velocity of blood flow is inversely proportional to the total cross-sectional area of blood vessels. As the total cross-sectional area of the vessel increases, the flow velocity decreases [49]. Accordingly, we observed that the mean blood-flow velocity and blood flow were significantly decreased, while the diameter increased in the 134 μM DK treatment group. Therefore, 134 μM DK treatment increased vessel diameter and regulated blood-flow parameters in the zebrafish model.

## 5. Conclusions

Herein, we revealed that ECE and DK effectively promoted endothelial-dependent NO production by activating the PI3K/Akt/eNOS pathway and [Ca^2+^]_cytol_ regulation. [Ca^2+^]_cytol_ levels are closely associated with the activation of AchM3R in EA.hy926 cells. Furthermore, in vivo experiments showed that DK treatment could promote vasodilation by increasing the DA diameter, further regulating blood-flow velocity in the zebrafish model. Based on the in vitro and in vivo experiments, we suggest that the ECE and DK possess superior vasodilatory effects and can be developed as suitable therapeutic agents.

## Data Availability

The data sets generated and/or analyzed during the current study are available from the corresponding author on a reasonable request.

## Supplementary Materials

The following are available online at https://www.mdpi.com/article/10.3390/biomedicines9040438/s1, Figure S1.
