# Supplementary material for: Ecklonia cava Extract and Its Derivative Dieckol Promote Vasodilation by Modulating Calcium Signaling and PI3K/AKT/eNOS Pathway in In Vitro and In Vivo Models
Source: Biomedicines. 2021 Apr 19;9(4):438. doi: 10.3390/biomedicines9040438 (PMC8073412; doi:10.3390/biomedicines9040438)
Supplement: Supplementary file 1 [file biomedicines-09-00438-s001.zip › biomedicines-1172639-supplementary.pdf]

Supplementary data- Material and method

Chromatography was performed on Agilent Technologies 1220 Infinity II LC with a column (poroshell 120 C18, 4.6*100 mm, 4μm). The mobile phase consisted of A; DW (+0.1% Formic acid), B; MeOH (+0.1% Formic acid) as followed: (0 min A; 63% B; 37%, 0-10 min A; 45% B; 55%, 10-12 min A; 63% B; 37%, 12-20 min A; 63% B; 37%). The gradient elution was performed as follows: the flow rate was 0.4 mL/min, and the injection volume was 1 mL. Detection was performed at UV length 230 nm.

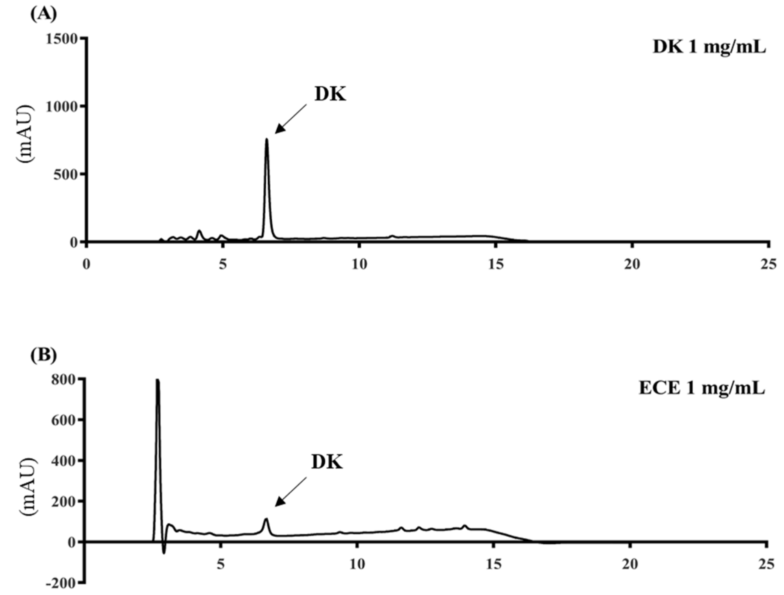

Figure S1. HPLC chromatography of (A) isolated DK (B) DK in ECE
